# Investigate the heterogeneity of colorectal cancer patients at the single-cell level prior to and subsequent to immunotherapy

**DOI:** 10.3389/fimmu.2026.1840165

**Published:** 2026-05-04

**Authors:** Heng Zhao, Gao Wu, Junhong Zhao, Qianwen Wang, Wenkang Cheng, Jie Li, Jin Yang

**Affiliations:** 1 Department of Laboratory Medicine, Hubei Provincial Clinical Research Center for central nervous system repair and functional reconstruction, Taihe Hospital, Hubei University of Medicine, Shiyan, Hubei, China; 2 Fujian Key Laboratory of Innate Immune Biology, Biomedical Research Center of South China, College of Life Science, Fujian Normal University, Fuzhou, Fujian, China; 3 Department of Cerebrovascular Disease Diagnosis and Treatment Center, Taihe Hospital, Hubei University of Medicine, Shiyan, Hubei, China; 4 Institute of Infection and Immunity, Taihe Hospital, Hubei University of Medicine, Shiyan, Hubei, China

**Keywords:** B cells, colorectal cancer (CRC), immune checkpoint blockade (ICB), single-cell sequence, T cells

## Abstract

**Background:**

Immune checkpoint blockade (ICB) has improved outcomes for a subset of colorectal cancer (CRC) patients, yet the cellular determinants that drive heterogeneous treatment responses remain insufficiently understood. The extent to which ICB reshapes immune populations across tumor, blood, and adjacent normal tissues at single-cell resolution is largely unexplored.

**Methods:**

We reanalyzed spatiotemporal single-cell RNA-sequencing data from CRC patients treated with Pembrolizumab or Sintilimab, profiling immune and stromal compartments across pre- and post-treatment tumor, blood, and normal mucosa. Major cell types and transcriptional programs were characterized using Seurat and Harmony. Treatment-associated cellular remodeling, differential gene expression, and response-related signatures were assessed in T cells and B cells. Cell-cell communication networks were inferred using CellChat.

**Results:**

ICB produced pronounced remodeling of tumor-infiltrating lymphocytes, particularly within T cell and B cell lineages. Responders (CR/PR) exhibited robust cytotoxic T-cell activation characterized by up-regulation of GNLY, GZMB, and CXCL13, whereas non-responders (SD) uniquely showed persistent overexpression of HSPA1B, a stress-response gene associated with tumor progression and immune suppression. Responders (CR/PR) exhibited robust cytotoxic T-cell activation, with increased expression of GNLY, GZMB, and CXCL13. In contrast, non-responders (SD) exhibited persistent overexpression of HSPA1B, a stress-response gene linked to tumor progression and immune suppression. B cells from responders demonstrated strong induction of TXNIP, a regulator of redox balance and inflammasome activity. This suggests a potential association between ICB treatment and a pro-inflammatory B-cell phenotype. Cell-cell communication analysis revealed that responders selectively enhanced CXCL, IL16, and CD22 signaling pathways. This indicates coordinated activation of a T-B-myeloid communication axis, which was absent in SD patients.

**Conclusions:**

This integrative spatiotemporal single-cell analysis reveals that effective ICB responses in CRC are associated with coordinated activation of cytotoxic T cells, TXNIP-associated transcriptional changes in B cells, and strengthening of CXCL-centered immune communication networks. In contrast, HSPA1B-high transcriptional states are associated with treatment resistance. These findings provide mechanistic insights into response heterogeneity and highlight potential candidate targets, such as HSPA1B and CXCR4-associated pathways for improving immunotherapy efficacy in CRC.

## Introduction

Colorectal cancer (CRC), being one of the most commonly encountered malignant tumors, makes up around 10% of all cancer cases and stands as the second most frequent cause of cancer-related deaths on a global scale (1). Despite advances in surgery, chemotherapy, radiotherapy, and targeted therapy, survival outcomes for patients with advanced or metastatic CRC remain poor. Immune checkpoint blockade (ICB), particularly inhibition of the PD-1/PD-L1 axis, has transformed treatment paradigms for several cancer types (2). In CRC, ICB has shown remarkable efficacy in tumors with microsatellite instability–high (MSI-H) or deficient mismatch repair (dMMR), where durable clinical responses are frequently observed. However, the vast majority of CRC cases—classified as microsatellite stable (MSS) derive limited benefit from PD-1 blockade, and even among MSI-H/dMMR tumors, substantial heterogeneity in therapeutic outcomes persists (3, 4). This variability underscores an urgent need to elucidate the immune mechanisms that determine ICB sensitivity or resistance in CRC (5). Previous studies have largely focused on immune states within tumors, identifying correlations between cytotoxic T-cell infiltration, neoantigen burden, and clinical benefit. Yet tumor immunity is orchestrated across multiple anatomical compartments, and systemic immune interactions may contribute to shaping treatment responses (6, 7). This perspective is further supported by growing evidence highlighting the clinical relevance of blood-based biomarkers in colorectal cancer. Recent studies have demonstrated that circulating DNA, RNA, and protein analytes in peripheral blood can serve as minimally invasive tools for disease monitoring and patient stratification (8). Therefore, integrating circulating blood with tumor and adjacent normal tissues may provide a more comprehensive understanding of immune dynamics and improve the translational relevance of single-cell analyses. A comprehensive spatiotemporal characterization of immune cells across tissues before and after ICB is therefore essential for understanding response heterogeneity, but such datasets have been scarce. Single-cell RNA sequencing (scRNA-seq) offers unprecedented resolution for deconstructing the cellular ecosystems underlying therapeutic responses (9). Recent work has applied scRNA-seq to CRC, revealing immune dysfunction, T-cell exhaustion, and stromal heterogeneity. However, key knowledge gaps remain. It is unclear how immune populations across tumor, blood, and normal mucosa change following ICB. The T-cell and B-cell subpopulations specifically distinguish responders from non-responders are also not well defined. In addition, how intercellular communication rewrite under effective ICB therapy remains unknown. To address these unresolved questions, we reanalyzed a recently published spatiotemporal single-cell dataset of CRC patients treated with Pembrolizumab or Sintilimab. By integrating high-resolution transcriptomic data from paired pre- and post-treatment tumor, blood, and normal tissues, we systematically dissected the cellular remodeling induced by ICB and identified molecular circuits that stratify clinical responders from non-responders.

Recent spatiotemporal single-cell studies have provided important insights into immune dynamics in colorectal cancer (CRC) under immune checkpoint blockade (ICB), particularly within tumor tissues. However, several limitations remain. First, most analyses have primarily focused on tumor-intrinsic immune features, with limited attention to coordinated immune responses across multiple compartments, including blood and adjacent normal tissues. Second, prior studies have largely emphasized individual immune cell populations, such as cytotoxic T cells, while the extent to which intercellular communication networks contribute to therapeutic heterogeneity remains incompletely characterized. Third, although B cells have emerged as important modulators of anti-tumor immunity, their transcriptional reprogramming and potential roles in ICB response in CRC are still not fully understood.

To address these gaps, we performed an integrative reanalysis of a published spatiotemporal single-cell dataset, focusing on cross-tissue immune coordination and cell–cell communication. Our study highlights multi-compartment immune remodeling, identifies coordinated T–B–myeloid signaling networks, and reveals a TXNIP-associated transcriptional program in B cells enriched in responders. These findings provide a systems-level perspective on immune remodeling and may help to better understand the heterogeneity of ICB responses in CRC.

## Material and methods

### Data collection and preprocessing

The single-cell sequencing data of colorectal cancer were obtained from GSE236581 in the GEO database. A total of 12 colorectal cancer (CRC) patients treated with immune checkpoint blockade (ICB), including Pembrolizumab and Sintilimab, were included in this study based on the availability of complete clinical information. Patients were classified into responders (complete response [CR] and partial response [PR]) and non-responders (stable disease [SD]) according to clinical response annotations provided in the original dataset. Detailed clinical characteristics of the included patients are summarized in **Table 1**.

**Table 1:** Clinical meta information.

Patient ID	Age	Gender	Cancer type	Tumor location	TNM	Tumor stage	dMMR/pMMR	MSI/MSS	POLE mutation	TMB
(Muts/Mb)	Tumor Regression Ratio	Response	TRG status	Treatment Regimen						
P01	51	Male	CRC	Descending colon	T4bN0M0	II	dMMR	MSS	Yes	Not available
P02	56	Male	CRC	Ascending colon	T4bN2M1	IV	pMMR	MSS	No	3.58
P04	47	Female	CRC	Ascending colon	T4aN+M0	III	dMMR	MSI	No	487
P08	52	Male	CRC	Low rectum	T3N1M0	III	dMMR	MSI	No	121.64
P11	65	Male	Duodenal carcinoma	Duodenum	T3N0M0	II	dMMR	MSI	No	1.78
P12	60	Male	CRC	Descending colon	T4bN2bM0	III	dMMR	MSI	No	114.72
P16	64	Male	CRC	Low rectum	T3N+M0	III	pMMR	MSI	No	13.66
P17	37	Male	CRC	Sigmoid colon	T4bN+M0	III	dMMR	MSI	No	67.7
P19	58	Male	CRC	Transverse colon	T3N0M0	II	dMMR	MSI	No	54.54
P20	48	Male	CRC	Ascending colon	T4bN+M0	III	dMMR	MSI	No	Not available
P23	37	Male	CRC	Transverse colon	T4bN2bM1	IV	dMMR	MSI	No	181.17

This cohort of samples was composed of responders categorized as “complete response” (CR, N = 7), “partial response” (PR, N = 2), and non-responders with “stable disease” (SD, N = 3).

Raw gene expression matrices were processed using the Seurat package (V4.3.0). Quality control filtering was performed to exclude low-quality cells: (1) fewer than 200 or more than 4,000 detected genes (2) the proportion of mitochondrial gene counts exceeded 5%. Cells with abnormally high gene counts were considered potential doublets and removed. In addition, erythrocyte contamination was filtered based on hemoglobin-related gene expression. After filtering, data were normalized and scaled using Seurat. Highly variable genes were identified for downstream analysis. To correct for batch effects across samples, the Harmony algorithm was applied (V0.1.1).

### Dimensionality reduction and cell clustering

Principal component analysis (PCA) was performed using the highly variable genes. The top principal components were selected for downstream analysis based on variance explained. Cells were clustered using a shared nearest neighbor (SNN) modularity optimization algorithm implemented in Seurat. Uniform Manifold Approximation and Projection (UMAP) were used for visualization of cell clusters. Cell-type annotation was performed based on canonical marker genes, including CD3D, CD3E for T cells; CD19 and CD79A for B cells; and LYZ for myeloid cells.

### Differential gene expression analysis

Differential gene expression (DEG) analysis was conducted using the “FindMarkers” function in Seurat. The Wilcoxon rank-sum test was used to identify genes differentially expressed between groups. P-values were adjusted for multiple testing using the Benjamini-Hochberg method. Genes with an adjusted p-value < 0.05 and |log2 fold change| > 0.25 were considered statistically significant. Comparisons were performed across multiple conditions, including treatment status (pre- vs. post-treatment), response groups (CR/PR vs. SD), and tissue types (tumor, blood, and adjacent normal tissues). Both horizontal (between response groups) and vertical (pre- vs. post-treatment) comparisons were conducted.

### Correlation analysis

The correlation between changes in immune cell proportions and tumor lesion variations was calculated using the Pearson correlation coefficient (r). Statistical significance was assessed where applicable.

### Cell-cell communication analysis

Cell-cell communication analysis was performed using the CellChat package (V1.6.1). Ligand-receptor interactions were inferred based on the CellChat database. Communication probability was computed to quantify interaction strength between cell types, and signaling pathway activity was compared across groups. Differential communication analysis was performed to identify pathways with altered signaling strength between responders and non-responders.

### Statistical analysis

All statistical analyses were performed using R software (V4.1.2). Unless otherwise specified, a two-sided p-value < 0.05 was considered statistically significant.

## Results

### Establish the microenvironment atlas for CRC

**Figure 1:**
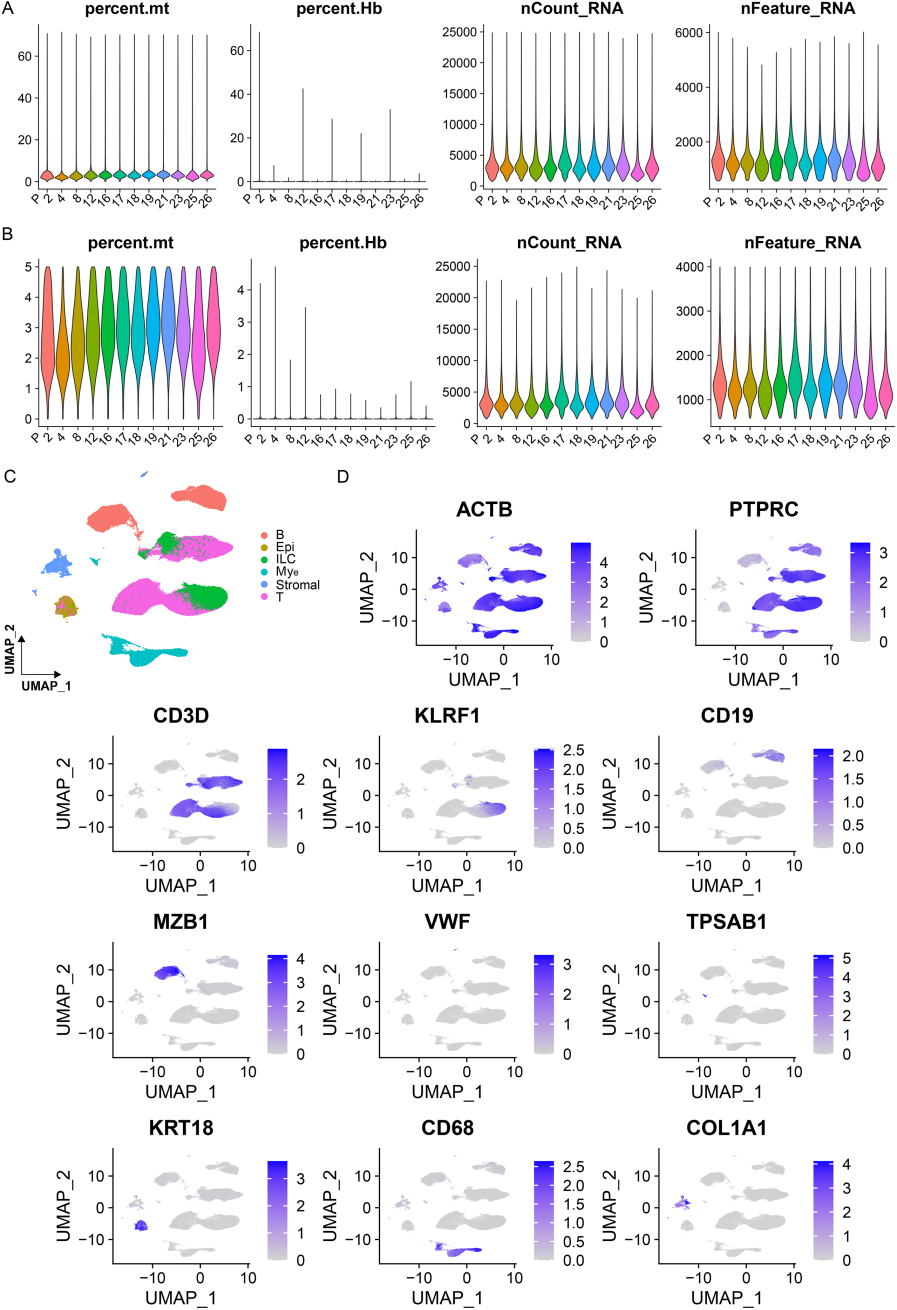
Overall quality control for single-cell data in colorectal cancer. **(A)** The proportion or expression profiles of mitochondria, red blood cells, nCount_RNA, and nFeature_RNA within the single-cell sequencing data. **(B)** Distributions of the same quality control metrics after filtering. Cells with fewer than 200 detected genes or with high mitochondrial content (>5%) were excluded. **(C)** UMAP visualization showing the distribution of annotated cell populations consistent with the original dataset. **(D)** UMAP feature plots showing the expression of representative marker genes across different cell populations. Each point represents a single cell.

The single-cell sequencing data of CRC were retrieved from GSE236581 (10). Twelve patients who had undergone treatment exclusively with Pembrolizumab and Sintilimab were selected, accompanied by samples furnished with comprehensive clinical information (encompassing details such as tumor alterations pre- and post-treatment, treatment effectiveness, and tumor mutational burden) (**Table 1**). This cohort of samples was composed of responders categorized as “complete response” (CR, N = 7), “partial response” (PR, N = 2), and non-responders with “stable disease” (SD, N = 3). Following an initial round of data collation, the proportions of mitochondrial genes, erythrocyte genes, and the total gene count were computed. Quality control procedures were implemented to sift out dead cells, doublets, erythrocytes, and other artifacts from the data (**Figures 1A, B**). Ultimately, the refined data set comprised 332782 cells (**Table 1**). The software tools Seurat and Harmony were utilized to integrate the single-cell data, cluster the cells, and identify marker genes (11, 12). Dimensional reduction of the entire cell population was achieved using umap to construct the single-cell atlas of CRC patients.

**Figure 2:**
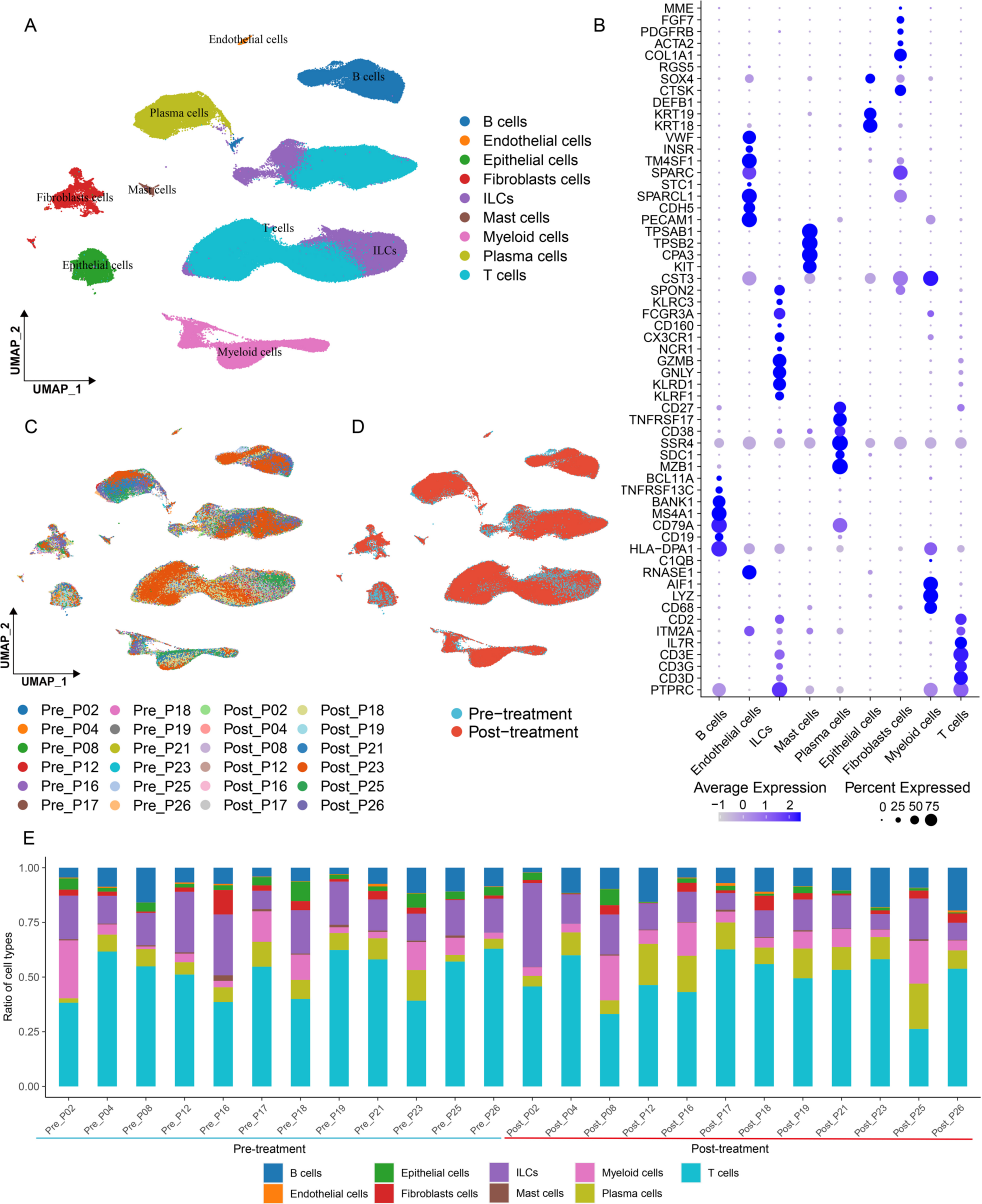
Single-cell atlas of colorectal cancer patients. **(A)** The umap plots were adopted to showcase the cell types as well as their distribution within colorectal cancer patients. **(B)** Bubble charts were employed to exhibit the expression profiles of the principal marker genes across different cells. **(C, D)** The umap plots were utilized to depict the cell types and their distribution scenarios in diverse samples, both prior to and following treatment. **(E)** Bar charts were applied to illustrate the proportion of cells in various samples.

The single-cell atlas of CRC patients incorporated T Cells, B Cells, ILCs, Mast Cells, Myeloid Cells, Plasma Cells, Fibroblasts Cells, and Epithelial Cells (**Figure 2A**). Specifically, T Cells were demarcated by genes like CD3D, CD3G, CD3E, and IL7R. In B Cells, genes such as CD19, CD79A, MSA4A1, and BANK1 exhibited high levels of expression. Innate lymphocytes were pinpointed by genes including KLRF1, KLRD1, GNLY, GZMB, and NCR1. Myeloid Cells were tagged by genes such as LYZ, C1QB, AIF1, and HLA-DPA1. In Mast Cells, genes like KIT, CPA3, TPSB2, and TPSAB1 were highly expressed. In Plasma Cells, genes such as MZB1, SDC1, SSR4, CD38, and TNFRSF17 were highly expressed. In Fibroblasts Cells, genes such as COL1A1, ACTA2, PDGFRB, and FGF7 were highly expressed. In Epithelial Cells, genes like PECAM1, CDH5, SPARCL1, and STC1 were highly expressed (**Figure 2B**) (10, 13, 14). In comparison to the original data, given that Plasma Cells represent the terminal differentiation stage of B Cells and their transcriptional mechanisms diverge substantially (15), Plasma Cells nested within B Cells were further dissected. The remaining cell clusters remained congruent with the cell types and distribution patterns in the original data (**Figure 1C**). Concurrently, umap was employed to visualize the expression profiles of select marker genes such as PTPRC (CD45), ACTB, CD3D, and KLRF1 across different cell clusters (**Figure 1D**), thereby bolstering the veracity of the cell clustering and marker gene identification processes.

On the basis of these findings, umap plots were deployed to exhibit the distribution and fluctuations of cells from diverse samples, both before and after treatment, in relation to different treatment outcomes and within various tissues (**Figures 2C, D**). It was observed that each sample encompassed all cell populations. A bar chart was devised to gauge the proportion of each cell population within each sample (**Figure 2E**). Cells before and after treatment were distinctly labeled. In paired samples, conspicuous changes in the cell populations were discernible, suggesting that ICB treatment is associated with alterations in different cell types.

### Examine the correlation between the alterations in tumor lesions and those in the proportion of immune cells

**Figure 3:**
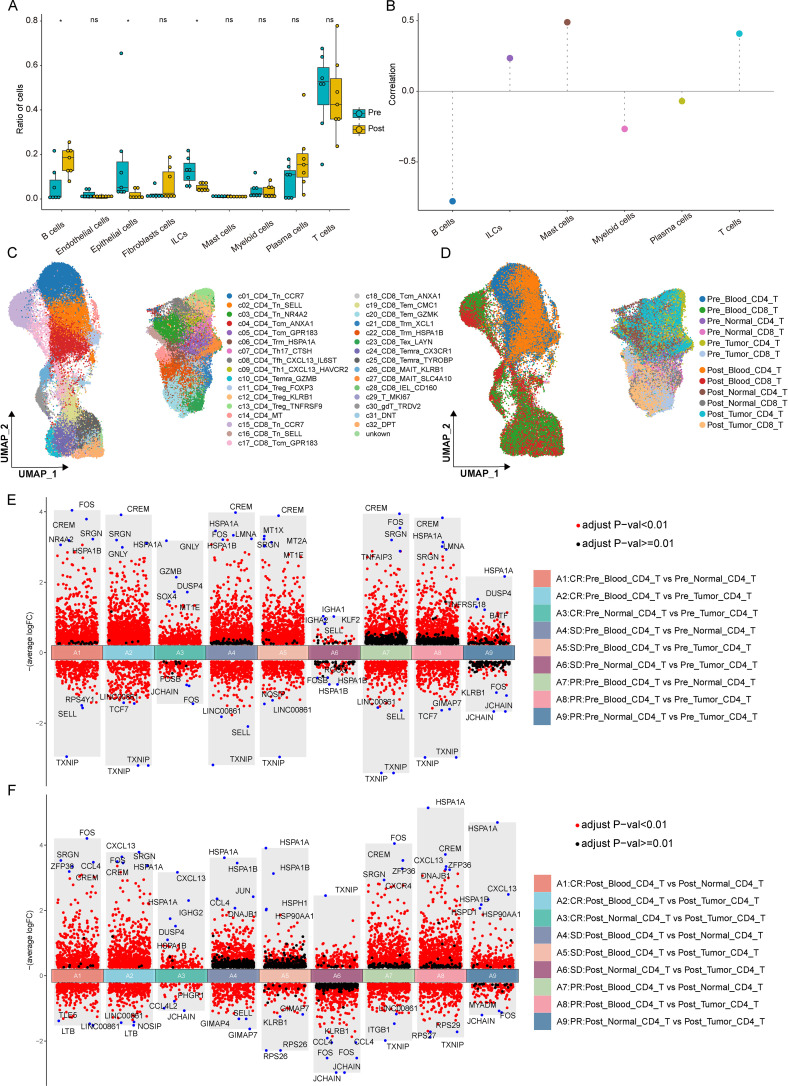
Correlation between the variations of immune cells and treatment outcomes, along with the alterations of CD4+ T cells in diverse tissues prior to and following treatment. **(A)** The variations in the proportion of each cell within tumors, both before and after treatment. **(B)** The correlation between the changes in immune cells within tumors and the relative changes of tumor biopsy lesions. **(C)** UMAP visualization of T-cell subclusters identified by unsupervised clustering. **(D)** The distribution of CD4+ and CD8+ T cells in umap plots, considering both before and after treatment as well as across different tissues. **(E)** The disparities in CD4+ T cells under different treatment outcomes and within different tissues prior to treatment. **(F)** The disparities in CD4+ T cells under different treatment outcomes and within different tissues after treatment. Differential gene expression analysis was performed using the Wilcoxon rank-sum test with Benjamini-Hochberg correction. Genes with adjusted p < 0.05 and |log2 fold change| > 0.25 were considered significant.

In order to ascertain the cell types meriting intensive investigation in CRC, a comparison was made regarding the disparities in cell proportions across different tissues prior to and following treatment. It was uncovered that conspicuous differences existed in B Cells and ILCs within tumor tissues (**Figure 3A**), whereas the discrepancies in other tissues were negligible (**Supplementary Figure 1A**). Consequently, a correlative analysis was carried out between the alterations of immune cells in tumors pre- and post-treatment and the fluctuations of tumor lesions. Specifically, the correlation between the two was gauged using the formula r=correlation(x, y), where x denotes the differential in the proportion of immune cells before and after treatment, and y represents the data on the changes of tumor biopsy lesions. This approach aimed to offer a more exhaustive evaluation of the most abundant immune cell types. It was revealed that T Cells and B Cells were selected for further analysis of tumor biopsy lesions were B Cells, Mast Cells, and T Cells (**Figure 3B**). Given the relatively scant quantity and proportion of Mast Cells, T Cells and B Cells were singled out as the focal points of subsequent research.

### Differences in T cells across diverse tissues and among various treatment outcomes

Unsupervised clustering analysis was carried out on T Cells to partition them into cell subgroups, which were found to be congruent with the T Cells subgroups in the original data. The distribution of each cell subgroup was visualized using umap (**Figure 3C**). Simultaneously, annotations were provided for pre- and post-treatment states, diverse tissue origins, as well as CD8 and CD4 T Cells, categorizing T Cells into groups like Pre-Blood CD8 T, Pre-Blood CD4 T, Pre-Normal CD8 T, and Pre-Normal CD4_T. Moreover, umap plots were generated to depict different groups before and after treatment and corresponding to various treatment effects (**Figure 3D**). Overall, it was observed that both CD8 and CD4 T Cells were present across different treatment effects and tissue types, attesting to the integrity of T Cells in this study.

To further delve into the multifaceted differences of CD8+ and CD4+ T cells in terms of treatment outcomes, tissue varieties, and pre- and post-treatment phases, a comprehensive integration of multiple conditions was implemented. These cells were bifurcated into CD8+ and CD4+ T cells, both pre- and post-treatment, and differential analysis was conducted across different tissues. Volcano plots were employed to showcase the top differentially expressed genes. Within each group, a horizontal comparison was first made to discern the differences among various treatment effects, followed by a vertical comparison to identify gene variances before and after treatment, thereby enabling a multifaceted comparison of multiple groups.

In the CD4+ group prior to treatment, the top differentially expressed genes varied substantially across different treatment effects and tissue types. In comparison with the SD group, it was noted that in the tumor tissues of the CR group, genes associated with cytotoxicity, such as GNLY and GZMB, were highly expressed, while in the tumor tissues of the PR group, the TNFRSF18 gene exhibited high expression levels (**Figure 3E**). Among these, GNLY and GZMB are pivotal molecules implicated in tumor treatment, playing crucial roles in cytotoxic lymphocytes and natural killer cells. Additionally, patients with low GZMB expression and elevated levels of immunosuppressive proteins PDL1 and IDO1 had the poorest prognoses (16). TNFRSF18 is correlated with the infiltration of cytotoxic T cells and the migration of NK cells in the tumor microenvironment, and the TNFRSF18 (GITR) agonist augments the antitumor response by potentiating CD8+CD4+ effector T cells (17). In the CD4+ group following treatment, when considering different treatment effects and tissue types, CXCL13 was highly expressed in the tumor tissues of the CR and PR groups compared with the SD group (**Figure 3F**). Compared with pre-treatment samples, CXCL13 expression showed an increasing trend after ICB therapy, suggesting a potential association with treatment response. Notably, HSPA1B expression remained relatively elevated in tumor tissues of the SD group, indicating a possible association with less favorable outcomes, and CXCL13 has been reported to be associated with improved efficacy of PD1 blockade (18).

Among these genes, CXCL13 is associated with T cell differentiation and also serves as a chemoattractant for B cells migrating to the germinal center (19, 20); the expression of HSPA1A and HSPA1B have been reported to be associated with tumor proliferation and progression (21). Following treatment, additional transcriptional changes were observed. In particular, CXCL13 and CCL4 were more highly expressed in responders, while HSPA1A and HSPA1B showed relatively higher expression in the PR and SD groups. These patterns suggest that differential transcriptional programs in CD8+ T cells are associated with distinct treatment outcomes.

**Figure 4:**
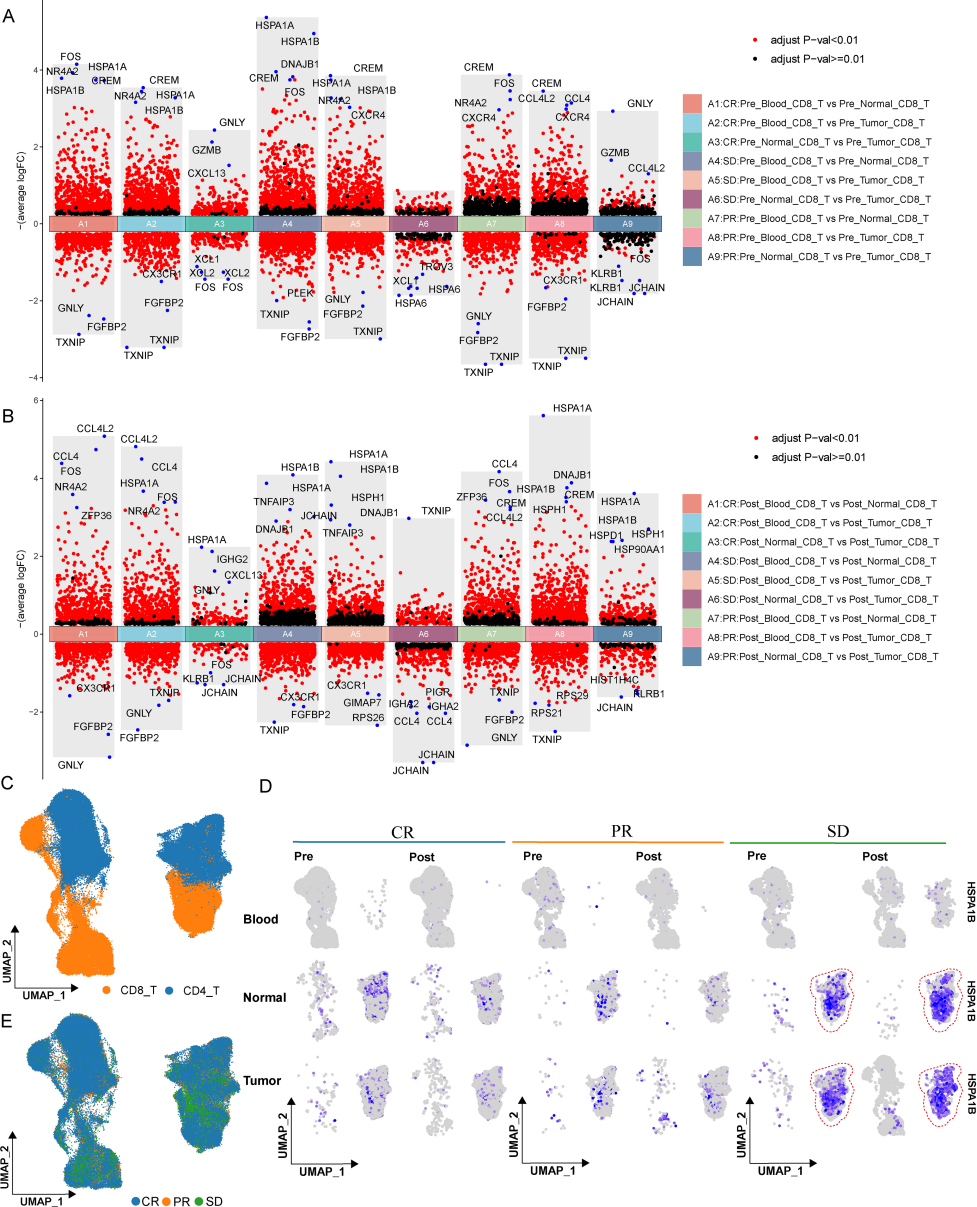
Transcriptional alterations in CD8+ T cells across treatment conditions. **(A, B)** Volcano plots showing differentially expressed genes in CD8+ T cells across response groups and tissue types before **(A)** and after **(B)** treatment. **(C)** UMAP visualization showing the distribution of CD4+ and CD8+ T cells. **(D)** UMAP feature plots showing the expression of HSPA1B across different treatment conditions, tissue types, and response groups. **(E)** UMAP plots showing the distribution of T cells across response groups. Differential gene expression analysis was performed as described in the Methods section.

To further probe the differences of specific genes across different treatment effects, tissue types, and cell categories, the expression of HSPA1B and GNLY genes was visualized using umap. Meanwhile, by referring to the cell distribution patterns of CD8, CD4 and different treatment effects (**Figures 4C, E**), the HSPA1B gene was compared across different groups. It was discovered that HSPA1B expression was primarily enriched in tumor and normal tissues of the SD group, suggesting a potential association with less favorable treatment response (**Figure 4D**). In contrast, GNLY expression was higher in tumor tissues of CR patients, consistent with its known association with cytotoxic lymphocyte activity (**Figure 4D**). These observations further support the link between cytotoxic signatures and treatment efficacy, while highlighting HSPA1B as a candidate marker associated with non-response.

### Differences in B cells across various tissues and among different treatment outcomes

**Figure 5:**
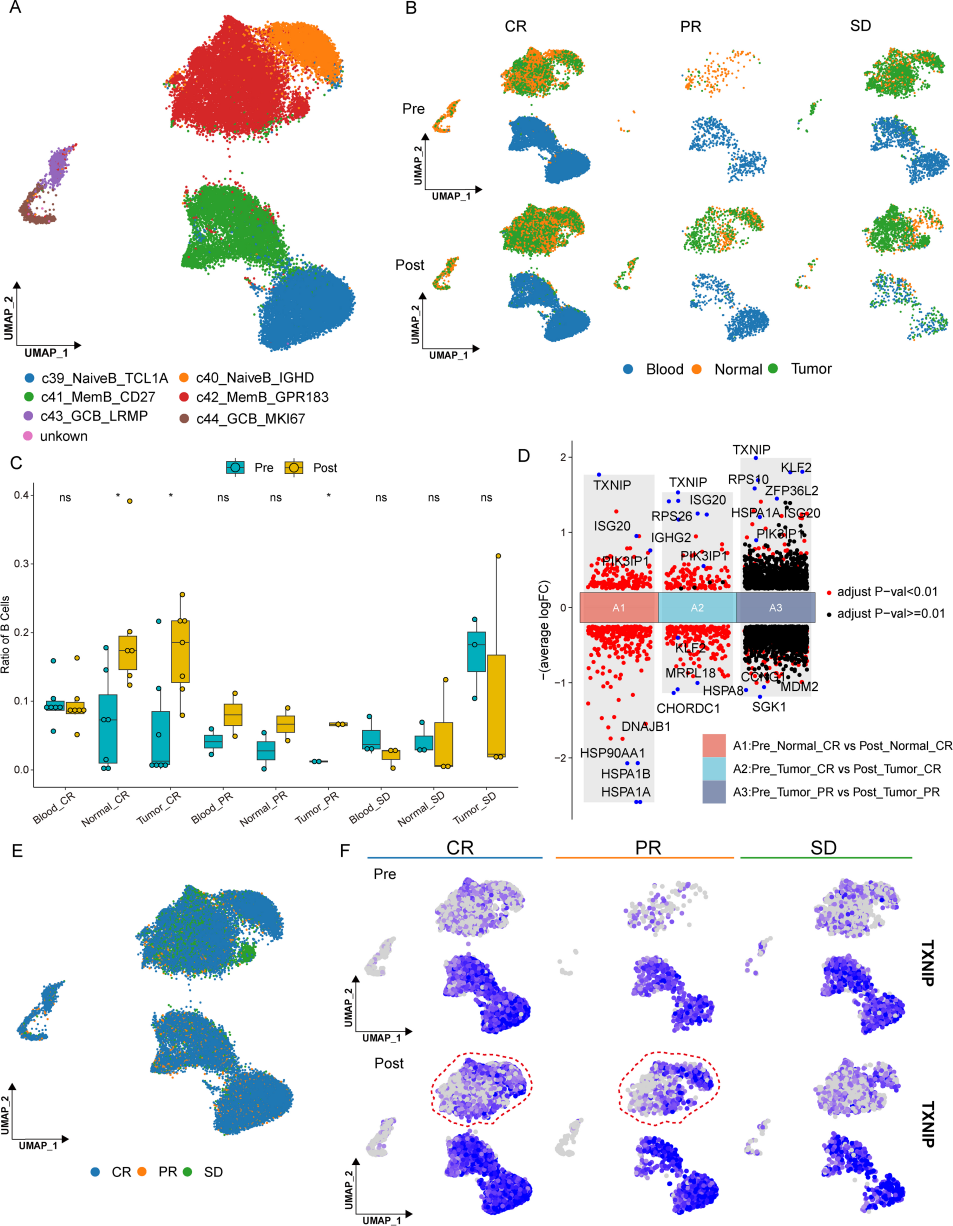
B-cell heterogeneity and transcriptional changes associated with treatment response. **(A)** UMAP visualization of B-cell subclusters identified by unsupervised clustering. **(B)** UMAP plots showing the distribution of B cells across treatment conditions, tissue types, and response groups. **(C)** Proportion of B cells across different tissues and treatment conditions. **(D)** Volcano plots showing differentially expressed genes in B cells before and after treatment. **(E)** UMAP plots showing the distribution of B cells across response groups. **(f)** UMAP feature plots showing the expression of TXNIP across different treatment conditions and response groups.

Unsupervised clustering analysis and subgroup partitioning were implemented on B cells. The B cell subgroups were demarcated by leveraging the marker genes sourced from the original dataset, and the resultant subgrouping was found to be in alignment with that of the original data. The B cell subgroups encompassed Naïve B_TCL1A, Naïve B_IGHD, Mem B_CD27, Mem B_GPR183, GCB_LRMP, and GCB_MKI67 (**Figure 5A**). Concurrently, umap plots were generated to depict the distribution of cells under varying treatment efficacies, across diverse tissues, and both pre- and post-treatment (**Figure 5B**). Moreover, a statistical analysis was conducted on the proportion of B cells within different groups. It was ascertained that significant discrepancies existed in normal and tumor tissues, both pre- and post-treatment, in the CR group, and likewise, notable differences were observed in tumor tissues post-treatment in the PR group. In contrast, no disparities were detected in blood, tumor, and normal tissues in the SD group (**Figure 5C**), suggesting a potential association between B-cell dynamics and treatment outcomes following ICB therapy.

To further scrutinize the corresponding genetic alterations in B cells pre- and post-treatment, differential analysis was carried out on the groups where differences had been identified. Upon obtaining the differential genes, corresponding volcano plots were crafted to showcase the top differential genes (**Figure 5D**). Among the identified genes, TXNIP and PIK3IP1 showed increased expression following treatment across multiple groups. TXNIP impacts the invasion of breast cancer cells via the TXNIP-HIF1α-TWIST signaling pathway and functions as a tumor suppressor in a plethora of cancers, including liver cancer, breast cancer, and lung cancer (22). The down regulation of the PI3K pathway, mediated by PIK3IP1, is anticipated to impede the progression of liver cancer cells (23).

Notably, TXNIP expression was further examined across treatment conditions. By integrating UMAP visualization and subgroup comparisons, we observed that TXNIP expression was enriched in B cells from the CR and PR groups after treatment (**Figures 5E, F**). This pattern suggests a potential association between TXNIP expression and ICB response. These findings highlight a possible role of B-cell transcriptional programs in treatment response and extend previous observations by implicating TXNIP as a candidate marker in this context.

### Variations in cell-to-cell communication among diverse cells prior to and following treatment

**Figure 6:**
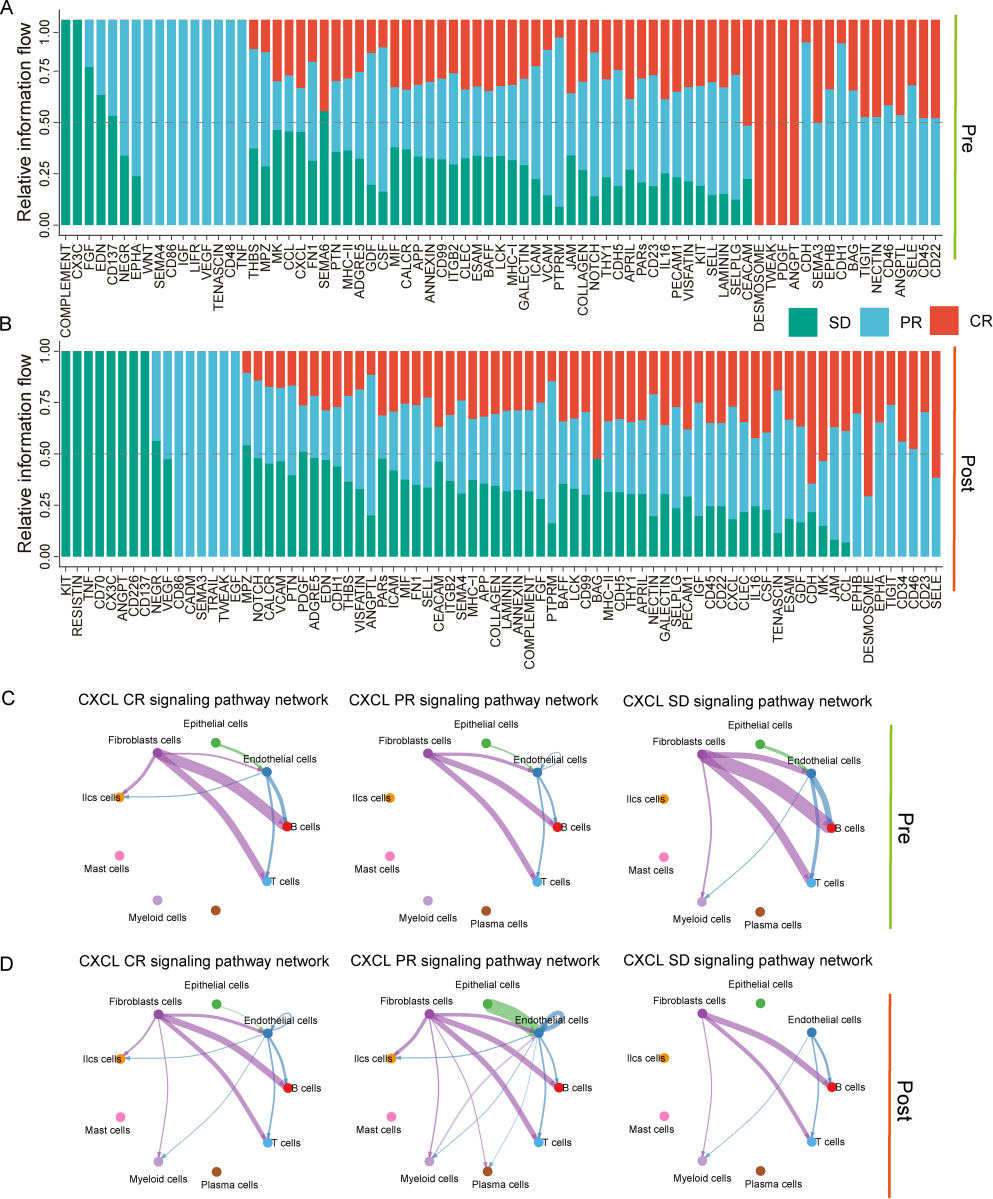
Variations in cell communication analysis among different treatment effects prior to and following treatment. **(A)** The variations in signal transduction pathways among different treatment effects before treatment were statistically analyzed. **(B)** The variations in signal transduction pathways among different treatment effects after treatment were statistically analyzed. **(C)** The disparities in the CXCL signal transduction pathway among different treatment effects before treatment were analyzed. **(D)** The disparities in the CXCL signal transduction pathway among different treatment effects after treatment were analyzed.

A total of 9 cell populations were encompassed in this current study, and intricate communication was present among these cell populations. To gain an understanding of the correlations between the receptors and ligands of cell populations and to dissect the alterations in communication among the entire cell populations, the CellChat tool was employed to carry out cell communication analysis on all cell populations (24).Differences in signaling pathways were observed among CR, PR, and SD groups before and after treatment. On the whole, a statistical analysis was performed on the signal transduction pathways of each of the CR, PR, and SD groups before and after treatment (**Figures 6A, B**). Notably, signaling pathways including CXCL, IL16, and CD22 showed increased activity following treatment. These pathways have been reported to be associated with immune cell interactions and tumor microenvironment dynamics, suggesting that intercellular communication patterns may differ across treatment outcomes. Specifically, CXCL, as a member of the chemokine family, is closely associated with the shaping of the tumor microenvironment (25); IL16 has the capacity to modulate the infiltration of tumor-associated macrophages (26); CD22 is typically associated with B cells and serves as a B cell-specific antigen, potentially playing a role in the treatment of hematological malignancies like B cell lymphoma (27).

An in-depth exploration was conducted on the signal transduction pathways of CXCL within different cells. Further analysis of CXCL signaling revealed that, prior to treatment, no substantial differences were observed among CR, PR, and SD groups. However, following treatment, increased CXCL signaling activity was observed in the CR and PR groups compared with the SD group (**Figures 6C, D**). In addition, genes such as CXCL2, CXCL3, and CXCR4 showed higher expression levels in myeloid cells and T cells after treatment (**Supplementary Figures 1D, E**). These findings suggest that CXCL-related signaling may be associated with coordinated immune responses in responders.

To further evaluate the clinical relevance of key genes identified in this study, we performed survival analysis using the TCGA colorectal cancer cohort. Patients were stratified into high- and low-expression groups based on gene expression levels. Kaplan-Meier analysis showed that higher TXNIP expression was significantly associated with improved disease-free survival, whereas HSPA1B exhibited a trend toward poorer survival outcomes, although this did not reach statistical significance (**Supplementary Figures 2A, B**). These results provide independent support for the potential clinical relevance of these genes.

## Discussion

Colorectal cancer (CRC) remains a significant global health burden, characterized by high incidence and mortality rates, with immunotherapy emerging as a transformative yet challenging modality (10). Immune checkpoint blockade (ICB), including agents like pembrolizumab and sintilimab, has demonstrated efficacy primarily in microsatellite instability-high (MSI-H) and deficient mismatch repair (dMMR) subtypes. However, microsatellite stable (MSS) CRC often exhibits resistance. This resistance is associated with immunosuppressive tumor microenvironments (TMEs), heterogeneous immune infiltration, and adaptive evasion mechanisms (28, 29). These limitations necessitate advanced single-cell analyses to unravel cellular dynamics and inform precision strategies.

In this study, we performed an integrative reanalysis of a published single-cell RNA-sequencing dataset to investigate immune heterogeneity in colorectal cancer (CRC) patients undergoing immune checkpoint blockade (ICB) therapy. Our analysis reveals that, consistent with previous reports, responders’ exhibit enhanced cytotoxic T-cell signatures. Importantly, beyond these established observations, we identify coordinated transcriptional and signaling changes across multiple immune cell types and tissues. This suggests that ICB response may involve system-level immune remodeling rather than isolated alterations within individual cell populations.

With respect to T-cell dynamics, our findings confirm that cytotoxicity-associated genes such as GNLY and GZMB are more highly expressed in responders, supporting their established association with effective anti-tumor immunity. These molecules are known to play central roles in cytotoxic lymphocyte-mediated tumor cell killing, and their elevated expression has been linked to improved clinical outcomes in multiple cancer types (30, 31). In addition, we observed increased expression of CXCL13 in responders following treatment. CXCL13 has been reported to be associated with tertiary lymphoid structure formation and enhanced responses to PD-1 blockade, suggesting that it may reflect an activated immune microenvironment (32, 33). Together, these observations are consistent with previous studies but further suggest that T-cell activation in responders may occur in coordination with broader immune changes across cell types.

Beyond T cells, our analysis highlights a potential role for B cells in modulating ICB response. We observed that TXNIP expression is enriched in B cells from responders following treatment, suggesting an association with specific transcriptional states. TXNIP has been implicated in redox regulation and cellular metabolic processes, and previous studies have suggested its involvement in tumor suppression pathways (34, 35). Although its role in B cells within the context of CRC immunotherapy remains unclear, our findings raise the possibility that TXNIP-associated transcriptional programs may reflect functional adaptation of B cells during ICB treatment. This observation extends prior studies by suggesting that B cells may contribute to treatment response beyond their traditional roles in antibody production, potentially through interactions with other immune cell populations (36, 37).

In addition, we identified differential expression patterns of stress-related genes such as HSPA1B, which was relatively elevated in non-responders. Members of the HSP70 family, including HSPA1B, have been associated with tumor progression and cellular stress responses (38, 39). While the precise role of HSPA1B in CRC immunotherapy requires further investigation, its enrichment in non-responders suggests that it may be associated with unfavorable immune states or resistance-related features (40). Taken together, these findings indicate that distinct transcriptional programs across immune cell populations may underlie differences in treatment outcomes.

A key contribution of this study lies in the analysis of intercellular communication networks. Using CellChat, we observed that signaling pathways such as CXCL, IL16, and CD22 are more active in responders following treatment. These pathways have been reported to be involved in immune cell recruitment, macrophage modulation, and B-cell function, respectively (41, 42). Rather than representing isolated signaling events, these findings suggest the presence of a coordinated T–B–myeloid communication axis. This network-level perspective provides additional insight beyond cell-type-specific analyses and supports the notion that effective ICB responses may be associated with integrated signaling across multiple immune compartments (43, 44).

Importantly, by integrating tumor, blood, and adjacent normal tissues, our study provides a multi-compartment view of immune remodeling in CRC. Compared with previous studies that primarily focused on tumor tissues, this cross-tissue approach highlights the potential contribution of systemic immune coordination to treatment response (45, 46). These findings suggest that immune dynamics beyond the tumor microenvironment may be relevant for understanding heterogeneity in ICB efficacy (47).

This study has several limitations. First, the analysis is based on a previously published dataset with a relatively limited sample size and heterogeneous clinical characteristics. In particular, cohort heterogeneity, including differences in treatment regimens, tissue sources (tumor, blood, and adjacent normal tissues), and sampling timepoints (pre- and post-treatment), may introduce potential confounding effects and partially influence the observed results (48). To mitigate this issue, we performed stratified analyses across response groups, tissue types, and treatment stages, and clarified these analytical strategies in the Methods and Results sections. Nevertheless, residual heterogeneity may still affect the interpretation of some findings. Second, the findings are derived from computational analyses and lack independent experimental validation (49). Third, the observational nature of single-cell transcriptomic data constrains mechanistic interpretation, and the identified associations do not imply causality. In addition, potential confounding factors, such as tumor heterogeneity and patient-specific variability, may influence the observed results (50).

Future studies are warranted to further validate these findings. Experimental approaches, including *in vitro* and *in vivo* functional assays, will be necessary to clarify the roles of candidate genes such as TXNIP and HSPA1B (51, 52). Moreover, integration with spatial transcriptomics and multi-omics data may provide deeper insight into the spatial organization and functional states of immune cells within the tumor microenvironment (53, 54). Prospective clinical studies with larger cohorts will also be important to evaluate the potential of these findings for biomarker development and therapeutic stratification (55–57).

In summary, our study provides a systems-level perspective on immune remodeling in CRC under ICB therapy. By integrating multi-tissue data and analyzing intercellular communication networks, we highlight the importance of coordinated immune interactions across multiple cell types. These findings contribute to a more comprehensive understanding of response heterogeneity and may inform future research on immunotherapy strategies in CRC (58).

## Data Availability

The data presented in the study are publicly available in the Gene Expression Omnibus (GEO) repository under accession number GSE236581.

## Supplementary material

Supplementary Figure 1 Differences in the Proportion of Cells within Blood and Normal Tissues and the Expression Status of Genes Associated with the CXCL Signal Transduction Pathway. **(A)** Disparities in the proportion of each individual cell within normal tissues. **(B)** Disparities in the proportion of each individual cell within blood tissues. **(C)** The expression of the GNLY gene under different treatment effects, both before and after treatment, and across different tissues was compared by means of umap plots. **(D)** The expression of genes related to the CXCL signal transduction pathway in diverse cells and under different treatment effects prior to treatment was presented. **(E)** The expression of genes related to the CXCL signal transduction pathway in diverse cells and under different treatment effects following treatment was shown.

Supplementary Figure 2 Survival analysis of HSPA1B and TXNIP in the TCGA CRC cohort. **(A)** Survival analysis of HSPA1B, **(B)** Survival analysis of TXNIP.

Supplementary Table 1 The cell count in each sample of CRC single-cell sequencing data before and after filtration.
