# Supplementary material for: Investigate the heterogeneity of colorectal cancer patients at the single-cell level prior to and subsequent to immunotherapy
Source: Front Immunol. 2026 May 4;17:1840165. doi: 10.3389/fimmu.2026.1840165 (PMC13180829; doi:10.3389/fimmu.2026.1840165)
Supplement: Supplementary Figure 1 — Differences in the Proportion of Cells within Blood and Normal Tissues and the Expression Status of Genes Associated with the CXCL Signal Transduction Pathway. (A) Disparities in the proportion of each individual cell within normal tissues. (B) Disparities in the proportion of each individual cell within blood tissues. (C) The expression of the GNLY gene under different treatment effects, both before and after treatment, and across different tissues was compared by means of umap plots. (D) The expression of genes related to the CXCL signal transduction pathway in diverse cells and under different treatment effects prior to treatment was presented. (E) The expression of genes related to the CXCL signal transduction pathway in diverse cells and under different treatment effects following treatment was shown. [file DataSheet1.pdf]

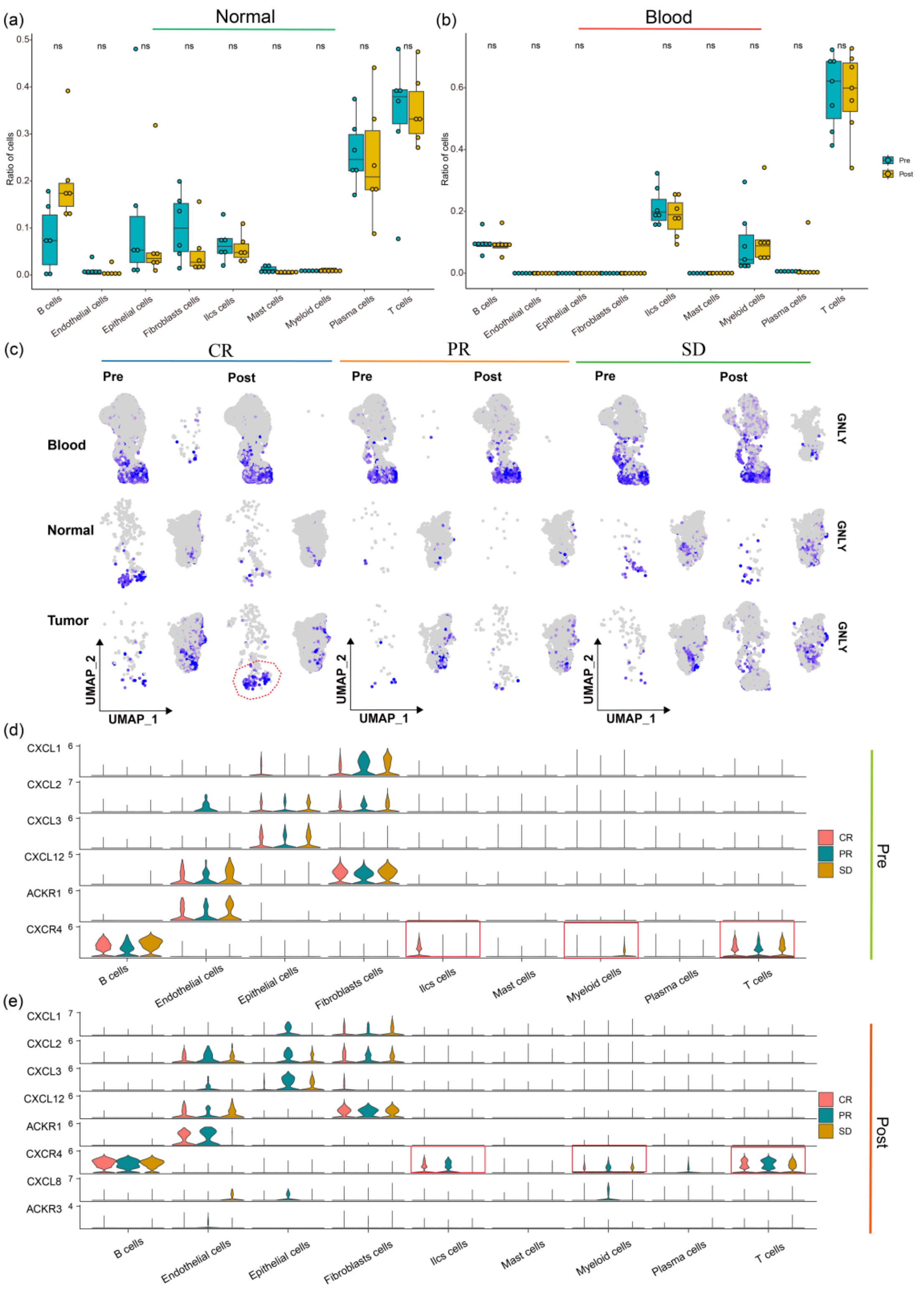

Figure S1: Differences in the Proportion of Cells within Blood and Normal Tissues and the Expression Status of Genes Associated with the CXCL Signal Transduction Pathway.(**a**): Disparities in the proportion of each individual cell within normal tissues.(**b**): Disparities in the proportion of each individual cell within blood tissues.(**c**): The expression of the GNLY gene under different treatment effects, both before and after treatment, and across different tissues was compared by means of umap plots.(**d**): The expression of genes related to the CXCL signal transduction pathway in diverse cells and under different treatment effects prior to treatment was presented.(**e**): The expression of genes related to the CXCL signal transduction pathway in diverse cells and under different treatment effects following treatment was shown.

TableS1: The cell count in each sample of CRC single-cell sequencing data before and after filtration

	P02	P04	P08	P12	P16	P17	P18	P19	P21	P23	P25	P26	SUM_data
raw_data	32000	48072	41768	26173	34513	24899	40868	31029	30437	30562	25458	38716	404495
clean_data	25855	44840	33854	22778	26914	19546	33602	22656	26524	24298	21633	30282	332782
