# Supplementary figures and images for: Investigate the heterogeneity of colorectal cancer patients at the single-cell level prior to and subsequent to immunotherapy
Source: Front Immunol. 2026 May 4;17:1840165. doi: 10.3389/fimmu.2026.1840165 (PMC13180829; doi:10.3389/fimmu.2026.1840165)

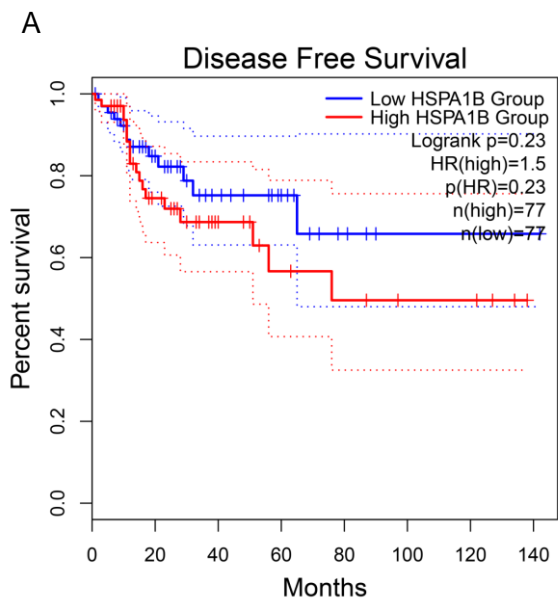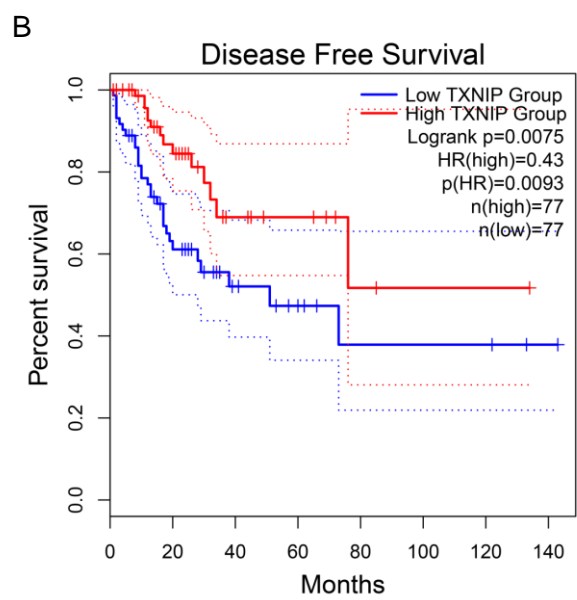

Supplement: Supplementary Figure 2 — Survival analysis of HSPA1B and TXNIP in the TCGA CRC cohort. (A) Survival analysis of HSPA1B, (B) Survival analysis of TXNIP. [file DataSheet2.pdf]
